# Radio Electric Asymmetric Conveyer (REAC) Biomodulation for Chronic Pain and Functional Improvement in Rheumatoid Arthritis, Lipedema, and Fibromyalgia: A Case Study on Anti-inflammatory, Circulatory, and Metabolic Optimization

**DOI:** 10.7759/cureus.74311

**Published:** 2024-11-23

**Authors:** Alessandra C Renck, Vania Fontani, Salvatore Rinaldi

**Affiliations:** 1 Diabetes and Endocrinology, International Scientific Society of Neuro Psycho Physical Optimization With Radio Electric Asymmetric Conveyor (REAC) Technology, São Paulo, BRA; 2 Research Department, Rinaldi Fontani Foundation, Florence, ITA; 3 Department of Regenerative Medicine, Rinaldi Fontani Institute, Florence, ITA

**Keywords:** chronic pain management, rheumatoid arthritis

## Abstract

This case report explores the use of radio electric asymmetric conveyor (REAC) technology for chronic pain management, functional limitations, and metabolic dysfunction in a 67-year-old female with rheumatoid arthritis, advanced lipedema, and fibromyalgia. The patient underwent three REAC protocols: Anti-cellulite treatment (ACT), circulatory optimization (CO), and metabolic optimization (MO), each targeting distinct pathophysiological aspects. The ACT protocol primarily addressed chronic inflammation contributing to pain and tissue dysfunction associated with lipedema. The CO protocol enhanced joint mobility by improving systemic and local blood flow, relieving joint stiffness due to rheumatoid arthritis. The MO protocol focused on metabolic restoration to counter chronic fatigue linked to lipedema and fibromyalgia.

SF-12 results showed a significant improvement in physical health index (50.88% to 68.16%) and mental health index (58.38% to 86.40%). Depression, anxiety and stress scale - 21 (DASS-21) scores indicated a decrease in the depression index (47.62% to 23.81%) and stress index (57.14% to 42.86%), with anxiety remaining stable at 23.81%. This case underscores REAC technology's potential as a non-invasive, multifaceted intervention addressing underlying inflammation, circulatory impairment, and metabolic dysregulation in complex chronic conditions, offering a valuable alternative to conventional treatments.

Following treatment, the patient reported substantial reductions in pain and physical discomfort, increased mobility, and enhanced energy and emotional well-being. Improvements began after ACT, with decreased pain and increased ease in daily activities. CO further supported joint mobility, allowing tasks like walking and stair-climbing with less strain. MO led to greater overall vitality and mood stability, enabling a more active lifestyle. No significant adverse effects were reported. This case underscores REAC technology's potential as a non-invasive, multifaceted intervention addressing the underlying inflammation, circulatory impairment, and metabolic dysregulation in complex chronic conditions, offering a valuable alternative to conventional treatments. Further research could clarify REAC's broader applications in similar multifactorial cases.

## Introduction

Chronic pain management in patients with multiple comorbidities, such as rheumatoid arthritis, lipedema [1], and fibromyalgia [2], presents a substantial clinical challenge. These conditions are characterized by systemic inflammation [3,4], neurophysiological disturbances [5,6], and metabolic dysfunction [7], often leading to debilitating symptoms that affect quality of life [8]. Conventional pharmacological treatments often provide only partial relief, and many patients fail to achieve significant improvements in functionality or well-being. Given these challenges, alternative therapeutic approaches are necessary to address the underlying mechanisms of pain, inflammation, and metabolic dysregulation.

REAC technology has emerged as a novel therapeutic tool, leveraging neuromodulation [9-11] and biomodulation [12-14] techniques to influence cellular and systemic processes. This case report investigates the application of REAC treatments through anti-cellulite treatment (ACT), circulatory optimization (CO), and metabolic optimization (MO) protocols [15-17] to manage chronic pain and associated symptoms in a 67-year-old female. The aim was to provide symptomatic relief and address the underlying factors contributing to her chronic conditions, resulting in functional improvement and enhanced well-being.

## Case presentation

Patient information

The patient is a 67-year-old woman with a complex medical history that includes long-standing rheumatoid arthritis (diagnosed at age 35), advanced-stage lipedema, fibromyalgia, and polycystic ovarian syndrome. She also experienced an anxiety-depressive disorder accompanied by persistent insomnia. She had undergone a total hysterectomy and a left ankle arthrodesis. At the time of presentation, her weight was 106.2 kg, with a body mass index (BMI) of 36.7, classifying her as obese. Her primary complaints included chronic pain, functional limitations due to restricted mobility, and mood disturbances. These issues significantly impacted her daily activities, leading to both physical and psychological distress.

**Figure 1:**
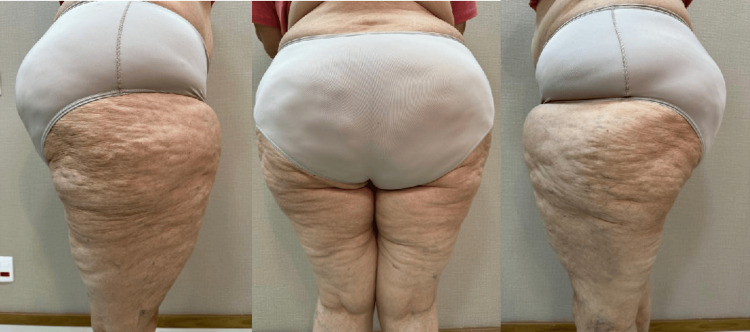
Advanced stage 4 lipedema in the lower extremities This figure illustrates the significant fat accumulation characteristic of advanced-stage 4 lipedema in the patient's hips and thighs. A) Right flank view; B) Posterior view; C) Left flank view. The abnormal fat deposits contribute to substantial physical discomfort and restricted mobility, exacerbating the patient's chronic pain and limiting daily activities. The lipedema is visibly pronounced, further complicating the patient's condition through increased inflammation and tissue dysfunction.

Her medical history highlighted the complex interaction between inflammatory and metabolic conditions that affected both her physical and mental health. Stage 4 lipedema caused abnormal fat deposits in her hips and thighs, contributing to considerable physical discomfort (Figure 1).

Rheumatoid arthritis further exacerbated her condition with joint pain and stiffness, particularly in the lower limbs. The presence of fibromyalgia compounded the complexity of her condition, adding widespread pain and fatigue to her symptoms.

Clinical finding

Upon assessment, the patient exhibited significant joint deformities, particularly in the lower extremities, consistent with the progression of rheumatoid arthritis. The lipedema was in an advanced stage, with substantial fat accumulation in the lower body, further contributing to her discomfort. The combination of her weight, BMI, and physical limitations severely impacted her mobility, making daily activities such as walking and climbing stairs challenging. Chronic pain, arising from rheumatoid arthritis, lipedema, and fibromyalgia, was a major factor limiting her functional capacity and also negatively impacted her mood and energy levels.

Diagnostic assessments

The diagnoses of rheumatoid arthritis, lipedema, fibromyalgia, and obesity-related chronic pain and insomnia were well-established based on her medical history and previous diagnostic evaluations. Prior pharmacological treatments had provided limited relief, and at the time of presentation, the patient was not undergoing any active rehabilitation or physical therapy. Given the complexity of her condition, a comprehensive approach was deemed necessary, prompting the initiation of REAC ACT, CO, and MO treatments to address the underlying physiological and metabolic dysfunctions contributing to her symptoms.

Therapeutic interventions

The patient's treatment plan utilized REAC technology through three specific protocols: ACT, CO, and MO. REAC biomodulation in these protocols works by modulating the bioelectrical activity of cells to restore normal cellular functions in areas of dysfunction. This technology employs weak radioelectric fields, conveyed asymmetrically to the treatment areas, to influence cellular responses, reducing inflammation, improving circulation, and restoring metabolic balance. Each protocol targets specific bioelectrical imbalances caused by inflammation, altered tissue perfusion, and metabolic dysfunction, addressing the underlying physiological disorders contributing to chronic conditions. The treatments were administered in 18 sessions for each protocol, with each session lasting approximately 15 minutes. The treatment was administered by placing the asymmetric conveyor probes (ACPs) on the patient's right and left quadriceps, which were connected to each other, and the REAC BENE 110 device (ASMED, Scandicci, Italy). The treatment parameters were pre-set and not modifiable, ensuring consistency and repeatability across sessions. The minimum interval between each session was at least one hour, with a maximum of four sessions per day. The administration followed a sequential approach, starting with the ACT protocol, followed by CO, and concluding with MO.

The ACT protocol was designed to reduce both acute and chronic inflammatory processes, which are also at the root of conditions like cellulite and lipodystrophy. The ACT protocol aimed to alleviate inflammation and improve local tissue function, particularly in the lower extremities, where the patient experienced significant discomfort.

The CO protocol aimed to enhance both systemic and local circulation, particularly targeting areas affected by joint pain and stiffness due to rheumatoid arthritis. By improving blood flow and reducing joint inflammation, CO contributed to better mobility and pain relief.

As with the ACT protocol, the ACPs were placed symmetrically on the quadriceps, connected to the REAC BENE 110 device, and administered according to pre-set parameters.

The MO protocol addressed the systemic metabolic dysfunction that was a key contributor to the patient's chronic pain, fatigue, and mood disturbances. The MO treatment sought to reduce systemic inflammation and enhance metabolic pathways, thereby improving energy levels and overall well-being. The treatment followed the same method of administration, with the ACPs positioned on the quadriceps and the device parameters fixed for precision and consistency.

This therapeutic strategy allowed for the targeting of underlying physiological mechanisms contributing to the patient's chronic pain, leading to significant improvements in her pain management, mobility, and quality of life.

Follow-up and outcomes

The patient experienced notable and progressive improvements throughout her REAC treatment regimen. Following the completion of the ACT protocol, she reported a subjective significant reduction in pain, particularly in areas affected by advanced-stage lipedema. This decrease in discomfort was attributed to the protocol's ability to reduce inflammation and improve tissue perfusion in the affected regions, which had previously caused substantial physical discomfort and limited mobility.

As the treatment progressed with the CO protocol, the patient observed marked improvements in joint mobility, especially in the lower extremities, where rheumatoid arthritis had caused significant stiffness and swelling. The CO protocol's enhancement of blood flow to the affected joints alleviated much of the swelling and inflammation, enabling the patient to regain greater functionality in daily activities such as walking and climbing stairs. This improvement in mobility also reduced the fatigue and strain she had previously experienced while performing basic tasks.

The MO protocol was pivotal in further enhancing the patient's overall well-being. By targeting systemic metabolic dysfunction, the MO protocol led to noticeable improvements in the patient's energy levels and emotional stability, which had been significantly impacted by fibromyalgia and polycystic ovarian syndrome. The reduction in systemic inflammation and metabolic imbalances helped manage her chronic pain more effectively and contributed to mood stabilization, allowing her to engage more actively in daily life with increased energy and a positive outlook.

The positive evolution of the patient's assessments in the SF-12 [18] and DASS-21 [19] tests demonstrates significant improvements in her physical and mental health indices. Specifically, the SF-12 physical health index [18] increased from 50.88% to 68.16%, and the mental health index [18] improved from 58.38% to 86.40%. Furthermore, the DASS-21 [19] depression index decreased from 47.62% (severe) to 23.81% (mild), while the stress index reduced from 57.14% (moderate) to 42.86% (moderate). The anxiety index remained stable at 23.81% (moderate).

By the end of the treatment regimen, the patient reported a subjective substantial improvement in overall pain levels, joint mobility, and functional capacity. She was able to perform daily activities with far greater ease and experienced a marked enhancement in her quality of life. The effectiveness of the REAC protocols in addressing the underlying inflammatory, circulatory, and metabolic imbalances contributing to her complex medical conditions was evident, demonstrating the potential of REAC technology as a multifaceted approach for managing chronic multifactorial conditions.

## Discussion

This case highlights the potential of REAC technology in managing complex, multifactorial conditions such as rheumatoid arthritis, lipedema [1], and fibromyalgia [4]. These chronic conditions are often intertwined with systemic inflammation, circulatory impairments, and metabolic dysfunction, creating significant challenges for conventional treatments. REAC technology addresses these issues through its biomodulation protocols, which work to restore cellular and systemic bioelectrical balance [9].

The ACT protocol effectively reduced localized inflammation and improved tissue function in the lower extremities, alleviating discomfort caused by advanced lipedema [15-17]. The CO protocol enhanced joint mobility and alleviated stiffness by improving blood flow in joints affected by rheumatoid arthritis [15-17]. Finally, the MO protocol provided systemic benefits by addressing metabolic dysfunction, leading to improvements in energy levels, emotional stability, and pain management [15-17].

Since the beginning of the treatment, the patient reported a significant improvement in her long-standing insomnia, which had previously impacted her quality of life. While the patient experienced transient insomnia during the last sessions of CO, this was not attributable to the treatment and was managed successfully with previously prescribed medication. The improvements in SF-12 [18] and DASS-21 [19] scores further support the efficacy of REAC treatments in enhancing both physical and mental health. This case underscores the value of a comprehensive therapeutic approach that targets the interconnected physiological mechanisms underlying chronic conditions. These promising results warrant further research to explore the broader applicability of REAC technology in similar cases.

## Conclusions

REAC technology, through its ACT, CO, and MO protocols, proved effective in managing chronic pain, functional limitations, and metabolic dysfunction in a 67-year-old female with rheumatoid arthritis, lipedema, and fibromyalgia. Each protocol addressed specific physiological imbalances, resulting in notable improvements in pain reduction, mobility, energy levels, and overall well-being.

The patient also experienced a marked improvement in her long-standing insomnia, highlighting the additional benefits of the treatments on her quality of life. The transient insomnia reported during the final CO sessions was not related to the treatment and was effectively managed with medication. The absence of significant adverse effects and the substantial improvements observed in both clinical assessments and patient-reported outcomes highlight the potential of REAC protocols as a non-invasive, multifaceted therapeutic option. Further studies are needed to validate these findings and explore the utility of REAC technology in managing complex, multifactorial chronic conditions.
